# Interdependence of metals and its binding proteins in Parkinson’s disease for diagnosis

**DOI:** 10.1038/s41531-020-00146-7

**Published:** 2021-01-04

**Authors:** Athira Anirudhan, Paramasivam Prabu, Jaya Sanyal, Tapas Kumar Banerjee, Gautam Guha, Ram Murugesan, Shiek S. S. J. Ahmed

**Affiliations:** 1 Faculty of Allied Health Sciences, Chettinad Academy of Research and Education CARE, Kelambakkam, 603103 India; 2 School of Medicine, Department of Neurology, University of New Mexico Health Sciences Center, University of New Mexico, New Mexico, USA; 3 Department of Anthropology, University of Delhi, Delhi, 110007 India; 4 Department of Neurology, National Neurosciences Centre, Kolkata, India; 5 Department of Neurology, Nil Ratan Sircar Medical College and Hospital, Kolkata, India; 6 Drug Discovery & Omics Lab, Faculty of Allied Health Sciences, Chettinad Academy of Research and Education, Kelambakkam, 603103 India

**Keywords:** Systems biology, Genetics

## Abstract

Metalloproteins utilizes cellular metals which plays a crucial function in brain that linked with neurodegenerative disorders. Parkinson’s disease (PD) is a neurodegenerative disorder that affects geriatric population world-wide. Twenty-four metal-binding protein networks were investigated to identify key regulating protein hubs in PD blood and brain. Amongst, aluminum, calcium, copper, iron, and magnesium protein hubs are the key regulators showing the ability to classify PD from control based on thirty-four classification algorithms. Analysis of these five metal proteins hubs showed involvement in environmental information processing, immune, neuronal, endocrine, aging, and signal transduction pathways. Furthermore, gene expression of functional protein in each hub showed significant upregulation of EFEMP2, MMP9, B2M, MEAF2A, and TARDBP in PD. Dysregulating hub proteins imprint the metal availability in a biological system. Hence, metal concentration in serum and cerebrospinal fluid were tested, which were altered and showed significant contribution towards gene expression of metal hub proteins along with the previously reported PD markers. In conclusion, analyzing the levels of serum metals along with the gene expression in PD opens up an ideal and feasible diagnostic intervention for PD. Hence, this will be a cost effective and rapid method for the detection of Parkinson’s disease.

## Introduction

Parkinson disease (PD) is one of the most common neurodegenerative disorders affects elderly populations. Over 10 million people are affected by PD world-wide^1^. The major hallmark of PD is an irreversible loss of dopaminergic neurons at substantia nigra region of the brain. Loss of dopaminergic neurons exhibit cardinal symptoms such as tremor, rigidity, and bradykinesia. Diagnosis of PD relies on medical history and radiological imaging^2,3^. Currently, there is no molecular markers are available to detect PD. Several epidemiological studies suggest multiple risk factors for PD, which includes aging, genetic mutation, and environmental exposure of metals and pesticides^4,5^. The genetic association of PD is well established, which reports PARK loci in most of the GWAS studies^6^. In contrast, the mechanism of metals and pesticide exposures contributing PD is largely unknown.

Emerging metallomic approach helps to investigate the role of metals in a biological system ^7^.

Metallomics is the large scale analysis of metals in cells, biofluids, or tissues. It uses high throughput the techniques such as atomic absorption spectroscopy, inductively coupled plasma mass spectrometry (ICP-MS), and inductively coupled optical emission spectrometry for the detecting and estimating the metal concentration in the biological system. Particularly, metallomic analysis of cerebrospinal fluid (CSF), serum, urine, and saliva has proven useful to identify metal biomarkers for several diseases. Additionally, metallomics provide a functional insight on metal interacting molecules such as DNA, RNA, protein, and metabolites. Of metal interacting molecules, metal-binding protein (metalloproteins) cover almost one-third of the human proteome^8^. Metal act as a cofactor that activates protein for basic molecular and cellular function^9^. Also, metal take part in stabilizing the conformational state of a protein in response to the substrate. Alternatively, metal generate oxidative stress, which serves as a major causative factor for most of the neurological diseases like Alzheimer’s, Parkinson’s, and Huntington’s disease^10,11^. In recent times integrating metallome with other omics data using systems biology has gained a better understanding of metal-mediated pathogenesis in various complex diseases^12,13^.

**Fig. 1:**
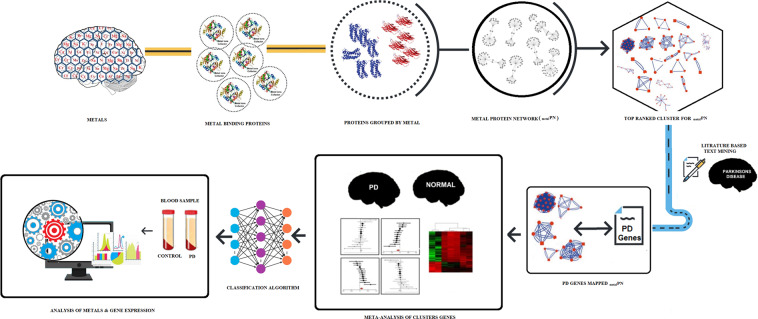
Systems biology framework describing the workflow of data collection, integration and analysis of metals and metal-binding proteins in Parkinson’s disease.

This study implement a systems biological approach (Fig. 1) to dissect the complexity of the metal-binding proteins involved in the pathogenesis of PD. Our approach integrates data mining of metal-binding protein, interactome, and meta-analysis to reveal the significant metal-associated protein hubs in PD. Additionally, we use classification algorithms in detecting highly contributing protein hubs, that classify PD from normal based on gene expression data. Further, the significant metal of each protein hub was measured in blood serum and CSF. Simultaneously, the gene expression of functionally important protein in each hub was analyzed, that reveals the interdependency between metal concentration and gene expression in PD pathogenesis which facilitate the application of metals and its genes as a biomarker in a clinical setting.

## Results

### Data collection and PPI

**Fig. 2:**
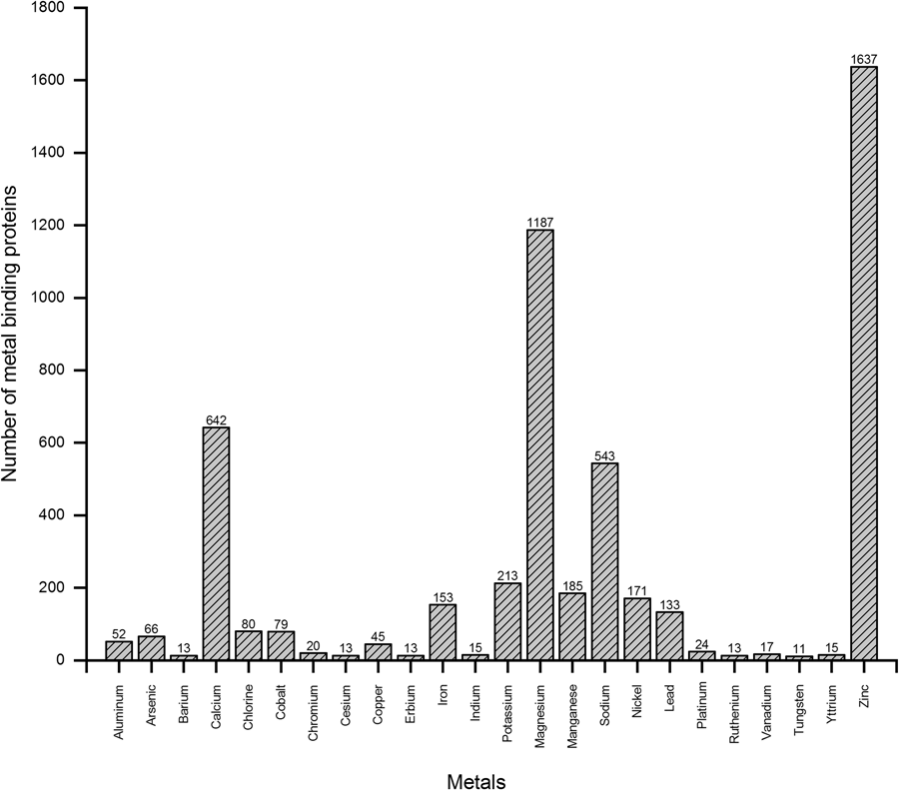
The Bar diagram indicating the number of metal-bounded proteins collected for each metal from the protein database.

**Fig. 3:**
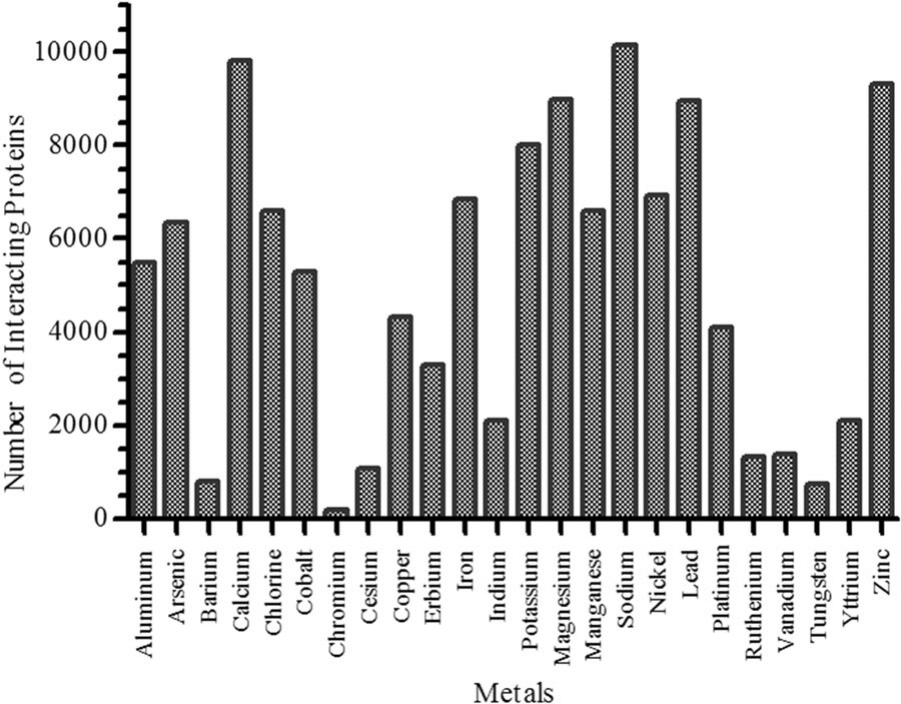
The Bar diagram indicate number of interacting protein in each _metal_PN.

We implement a systems biological workflow (Fig. 1) to illustrate the mechanism of metal mediating metalloprotein, a causative factor for PD. The metal-binding proteins were collected from various biological databases and curated to have 41 metals that bounded to 5368 proteins. Among which twenty-four metal-binding proteins groups were selected containing a minimum of ten proteins for each metal. For instance, zinc acts as a cofactor for 1637 proteins, whereas tungsten was noticed with minimum of 11 proteins (Fig. 2). Each metal-binding proteins group was subjected to protein–protein interaction to construct metal protein network (_metal_PN). For instance, copper was the cofactor for 45 proteins that form copper _metal_PN containing 4384 interacting proteins (see Supplementary Fig. 1). Overall, 24 _metal_PNs were constructed, which explicates the complex behavior of the metalloproteins with its interacting proteins (Fig. 3). Of every 24 _metal_PNs, 24 randomized, and 24 molecular-pathway networks were generated and subjected to topological analysis as described under the methodology section. Seven important topological parameters were determined for all 72 networks, which describes their network properties (see Supplementary Table 1).

### Principal component analysis (PCA), clustering, and text mining

An unsupervised PCA was performed to check the grouping ability of 24 _metal_PNs with randomized and molecular-pathway networks based on the topological parameters. Two distinct classification groups were noticed in the PCA model (see Supplementary Fig. 2) group 1—random networks; group 2—molecular pathway networks and _metal_PNs. The principle component 1 (PC1) accounts for 56.6% and component 2 accounts for 15% of the total variance. Together both the components explain around 71.6% of the data variability. All 24 _metal_PNs were grouped with 24 molecular-pathway networks indicating that the constructed _metal_PNs are biologically meaningful. Whereas, the 24 randomized networks were distinctly separated in PCA plot (see Supplementary Fig. 2).

Further, the top-ranked protein hub was identified for each _metal_PN using ClusterONE algorithm. Among 24 over-representing hubs, 18 showed the association with PD containing minimum of one text-mined PD protein. For example, the hub derived from copper _metal_PN contains six proteins of which all were previously reported in PD confirming the copper association with PD.

### Meta-analysis

To determine the regulation of selected 18 hubs in PD brain and blood tissue. The gene expression data of brain (GSE7621, GSE8397, GSE19587, GSE20141, GSE20146, GSE20163, GSE20164, GSE20168, GSE20186, GSE20291, GSE20292, GSE20295, GSE28894, and GSE49036) and blood (GSE6613, GSE22491, GSE54536, and GSE72267) were retrieved from NCBI-Geo Dataset and EBI-ArrayExpress databases, covering the inclusion criteria of our meta-analysis. The dataset include (normal vs. PD) 291 vs. 304 and 53 vs.104 participants in brain and blood, respectively. The meta-analysis of 302 proteins encoding genes of 18 hubs were analyzed using integrative meta-analysis of gene expression (INMEX), considering the primary goal to identify differentially expressed genes between control vs. PD in brain and blood, respectively. Of 302 genes, 75 were significantly differentially expressed (39 over and 36 were under expressed) in PD brain, which attributed to 16 _metal_PN hubs. Similarly in blood, five genes were upregulated and 86 were downregulated, that attributed to 17 _metal_PN hubs. The genes of these hubs were mapped with their appropriate gene expression data of the brain (normal = 291; PD = 304) and blood (normal = 53; PD = 104) and subjected to the classification algorithm.

### Classification algorithm based _metal_PN classification

Thirty-four distinct classification algorithms were implemented on each hub with their proteins as features, mapped with corresponding gene expression of brain and blood as instances. For example, the copper hub contains six proteins as features that mapped with appropriate gene expression from the merged brain datasets of 595 instances, normal = 291, PD = 304. Similarly for the blood, the copper hub with the same features contains (53 normal and 104 PD 157 instances). The classification ability of predicting normal or PD by each algorithm for brain and blood was evaluated by accessing the output based on sensitivity, specificity, accuracy and Matthews’s correlation coefficient. Based on the performance, top five hubs showing better accuracy between 34 algorithms were selected. For example in brain, iron showed better performance with accuracies ranged between 50 and 91% in all 34 algorithms, whereas cesium hub showed least accuracies between 44 and 56%. Of analyzed hubs, aluminum, calcium, copper, iron, and magnesium were over represented in most of the algorithm and commonly noticed in both brain and blood contributing towards better classification of PD from normal (see Supplementary Fig. 3). Further, the pathway enrichment analysis of 132 proteins of these five hubs showed the association with wide spectrum of molecular pathways related to PD (see Supplementary Fig. 4 and Supplementary Table 2). Particularly, the calcium, copper, iron, and magnesium hubs were commonly regulates 29 molecular pathways (see Supplementary Fig. 5). These pathways attributed to environmental information processing, immune, neuronal, endocrine, aging, and signal transduction mechanism. Subsequent prioritization analysis of proteins in each metal hub suggest EFEMP2, MMP9, B2M, MEAF2A, and TARDBP are functionally important in aluminum, calcium, copper, iron, and magnesium hub, respectively.

### Metal concentration and gene expression

**Fig. 4:**
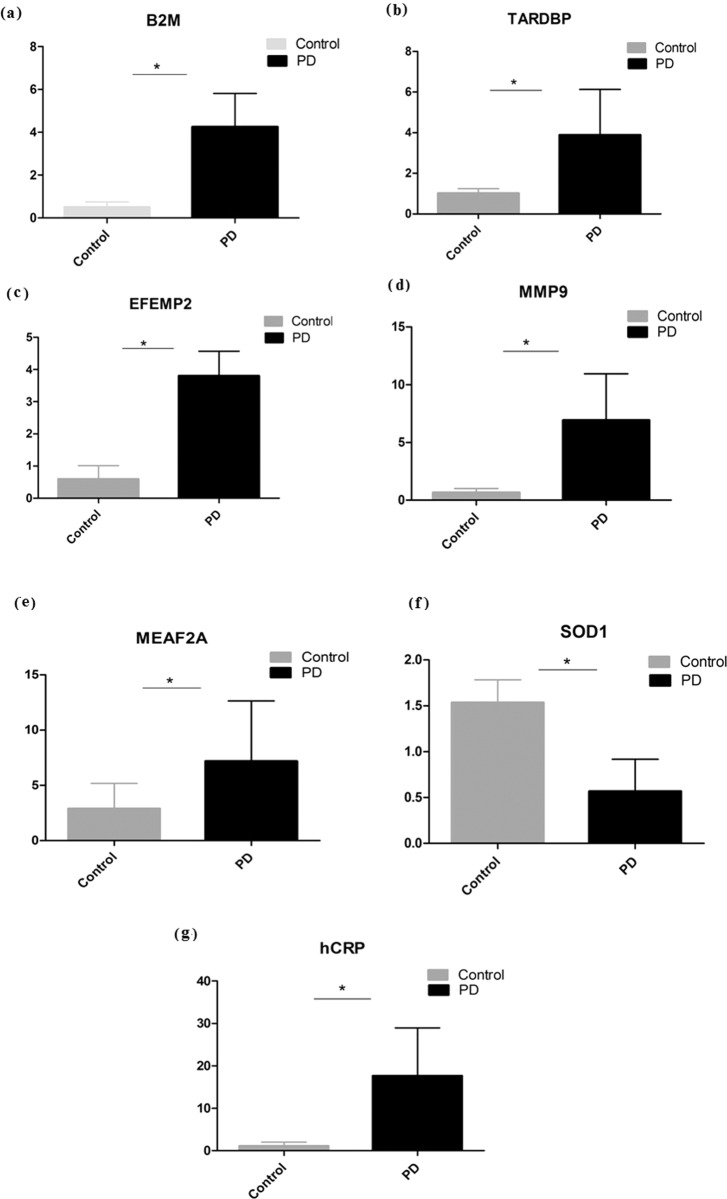
Quantitative real-time gene expression analysis of hub proteins encoding genes in PBMCs from control vs. PD. **a** B2M, **b** TARDBP, **c** EFEMP2, **d** MMP9, **e** MEAF2A relating copper, magnesium, aluminum, calcium, and iron, respectively. The gene expression of **f** SOD1, and **g** hsCRP are the markers for PD. Bars represent the Average, standard deviation (SD) with *P value < 0.05.

Estimating these metal concentrations in serum and CSF using atomic absorption spectroscopy (AAS) showed a significant increase in serum aluminum, calcium and magnesium and significant decrease in copper and iron in PD (Table 1). In CSF, significant decrease in aluminum, copper and iron and significant increase in calcium and magnesium were noticed in PD compared to control. The concentrations of analyzed metals were expressed in µg/L with their mean and standard deviation (Table 1). Similarly, the gene expression analysis of EFEMP2, MMP9, B2M, MEAF2A, TARDBP, and hsCRP with qPCR showed significant upregulation, whereas SOD1 was significantly down regulated in PD compared to control (Fig. 4a–g). Further, the interdependency between serum and CSF metal concentrations showed significant change in correlation pattern suggest altered ion transports across blood brain (BB) and blood CSF (BC) barrier in PD (see Supplementary Table 3). Similarly, significant association between metal concentrations with gene expression showed influence of altered serum metals regulates PD hub genes. Interestingly, these metals noticed regulating the gene expression of known oxidative and inflammatory markers (SOD1 and hsCRP) of PD (see Supplementary Tables 4, 5).

**Table 1:** Metal concentrations in serum and CSF determined using atomic absorption spectroscopy showing significant change in aluminum, calcium, copper, iron, and magnesium in PD compared to control.

	SERUM (Control = 87; PD = 87)			CSF (Control= 42; PD = 42)		
Metal	Control (µg/L)	PD (µg/L)	*P*-value	Control (µg/L)	PD (µg/L)	*P*-value
Aluminum	4.11 ± 1.52	4.755 ± 1.4	<0.02^a^	3.12 ± 0.886	2.43 ± 1.42	<0.03^a^
Calcium	64,825 ± 1514	72,303 ± 1720	<0.001^a^	26,291.15 ± 5411.9	28,447.38 ± 3378.10	<0.03^a^
Copper	1133 ± 127	909 ± 337	<0.001^a^	27 ± 4.78	24.4 ± 8.06	<0.029^a^
Iron	1265 ± 439	1091 ± 434	<0.009^a^	221 ± 28	172 ± 83.8	<0.002^a^
Magnesium	20,031 ± 1923	21,276 ± 1915	<0.0006^a^	23,030 ± 2659	25,466 ± 3512	<0.00055^a^

^a^Statistical significance (*P*-value ≤ 0.05**)** was calculated using *t*-test.

## Discussion

Metal-binding proteins were collected from various biological databases and grouped based on the metal cofactor to have 5368 proteins. For instance, copper acts as a cofactor for 45 proteins (Fig. 2). Of these, α-synuclein is one of the copper-binding proteins considered as a hallmark for PD, localized at presynaptic terminals of neurons. Similar to copper, there are other metals such as aluminum, calcium, chromium, iron, magnesium, lead, and zinc act as a cofactor for several proteins involved in various pathological process of PD. These proteins were grouped based on their bounded metal and subjected to protein-protein interaction to construct metal protein network _metal_PN. Further, the top-ranked hub of each _metal_PN was selected which reveals its core functional mechanism in the network. For example, copper _metal_PN was dissected into six hubs among which the top-ranked hub contains MAP1S, B2M, CD1A, CD1B, CD1D, and CD1E proteins (see Supplementary Fig. 4). Of 24 identified top-ranked hubs, 18 hubs contain minimum of one text-mined PD proteins. In copper hub (see Supplementary Fig. 4), all proteins (MAP1S,B2M, CD1A, CD1B, CD1D, and CD1E) were previously reported in PD^14,15^. Furthermore, the meta-analysis of 18 hubs showed 16 hubs in the brain and 17 in blood were dysregulated in PD. In copper hub, B2M and MAP1S were differentially regulated in both the brain and blood confirming its impairment in PD. B2M is the members of major histocompatibility complex-1 (MHC-1) molecules, widely expressed in various regions of the human brain. Also, it is well established that MHC-1 in neurons play a vital role in synaptic input, synaptic plasticity, and regulates axonal regeneration^16,17^. The expression of MHC-1 is appeared to be influenced in various neuronal pathways of the brain. Further, MHC-1 is involved in cerebellum motor learning and cortical synapse function^16–18^. Several reports suggest altered MHC-1 expression related to neuro-inflammatory mechanism that implicates the immune mediated neurodegenerative process in diseases, including PD^19,20^. Evidence suggests that B2M has a strong association with complex neurodegenerative diseases such as Alzheimer’s and Parkinson’s disease^21^. In addition neuronal microtubule-associated protein (MAP1S) in copper hub involved in axonal transport, regional specialization, and synaptic function^22^. Similar to copper hub, there are other metal hubs noticed to have significant functional association with PD. However, we have tried to explain the importance of our results using the copper hub.

Thirty-four classification algorithms were implemented on each hub to identify the hub showing better classification ability that differentiate PD from normal based on gene expression data. Of analyzed hubs, aluminum, calcium, copper, iron, and magnesium showed better performance in both the tissue for most of the algorithms (see Supplementary Fig. 3). The proteins of these hubs were subjected to enrichment analysis, which showed involvement in immunological, neuronal, endocrinal, and aging process that linked with PD pathogenesis. The proteins of copper hub showed a significant involvement in tight junction, amebiasis and MHC signaling pathways. Tight junctions between the cells contribute fundamental structure of blood-brain barrier (BBB). BBB play a crucial role in maintaining a stable environment of the central nervous system. Altered tight junctions and BBB disruption has been reported in the PD pathogenesis^23^. Overall, our pathway analysis paves a way to understand the role of five significant metal hubs in Parkinson’s disease (see Supplementary Table 2).

Dysregulating hub proteins imprint the metal availability in biological system. Thus, metallomic approach was carried out to estimate the concentrations of aluminum, calcium, copper, iron, and magnesium in blood serum and CSF. The analysis of metals showed significant variation in serum and CSF of PD (Table 1) compared to control. All analyzed metals of controls were in reference range of previously published literature^24^. Although, we cannot rule out importance of hub proteins as a possible biomarkers for PD. The hub proteins were prioritized to select functionally important protein from each hub which showed differential regulation in peripheral blood mononuclear cell (PBMC) of PD compared to control (Fig. 4). Mohit Kumar et al.^25^, observed the significant decrease in serum iron levels in PD patients. Similarly, Logroscino et al.^26^, and Hedge et al.^27^, noticed the decreased serum iron concentration and demonstrated its role in PD compared to control. In addition, Jaya Sanyal et al.^24^, showed the decreased copper and iron concentrations in CSF. Similar, results were noticed in this study confirming the importance of these metals in serum and CSF of PD pathology. Also, significant change in serum-CSF metal interdependency of aluminum, calcium, copper, and magnesium between CSF and serum in PD, suggest the impairment of metal transport in BBB and BCB (see Supplementary Table 3).

On the other hand, metal-gene interdependency (see Supplementary Table 4) explores the influence of metals on gene expression in PD. In copper hub, the expression B2M protein encoding gene with the copper concentration showed negative correlation which suggests that the decreased copper concentration increases the B2M gene expression in PD. Increased B2M has been observed in patients with Alzheimer’s disease^28^ dementia^29^, Parkinson’s diseases^30^, and Schizophrenia patients^31^. Also, Zhang et al. showed B2M induced rat causes cognitive impairments, depression, and anxiety that are closely linked with Parkinson’s disease symptoms. Similar metal-gene interdependency was noticed between magnesium-TARDBP, iron-MEAF2A, aluminum-EFEMP2, and calcium-MMP9, suggesting its possible influence in PD. In addition the association between metal with SOD1 and hsCRP marker showed negative correlation which suggest decreased copper concentration increase the expression of SOD1 and hsCRP in PD (see Supplementary Table 5). Also, comparing the interdependency between B2M (copper hub protein) with SOD1 and hsCRP exhibits positive correlation (see Supplementary Table 6). Trist^32^ reports that the maturation of SOD1 is blocked by depletion in copper level in brain specific region of PD^33,34^. This lead to decreased antioxidant property of SNc dopaminergic neurons against oxidative stress. Hence, inadequate copper concentration likely lead to the accumulation of copper deficient wild type SOD1 in brain. Similar results also obtained in our study that supports the decreased copper concentration causes over expression of B2M, SOD1, and hsCRP leading to PD pathogenesis.

Overall our finding provides an insight on the metals and metal-binding proteins in PD pathogenesis. However, our studies have few limitations such as (1) The samples included in this study are south Indian population. Adding the sample from various ethnic background and other neurodegenerative diseases will give more support on reliability of identified metal and gene expression based biomarker for PD. (2) Our study estimated the presence of interdependency between metal and gene expression, having protein expression will provide more information on this association. On the other hand, the strength of our study need to be acknowledged that, (1) This is the first study reporting the metalloprotein network to describe the influence of metal-binding protein in PD. (2) this study integrates metallomic and molecular technique to identify an feasible biomarker from serum. (3) Also, this study provide utilization of classification algorithm, which further reduce complexity of PD diagnosis.

In summary, our study helps to understand the role of metals and its binding protein in Parkinson’s disease. The significant changes in CSF and serum level of metals opens up an ideal and feasible diagnostic intervention for PD. Similarly, the interdependency between serum and CSF metal concentration of aluminum, calcium, copper, and magnesium shows significant changes in PD. This alteration makes dysregulation in ion transport across the blood-brain and blood-CSF barrier, which may throw light towards PD diagnosis. Although, the analyzed metals of CSF and serum showed important contribution in PD pathogenesis our potential interest is to identify a feasible markers for diagnosis. Considering the feasibility of both the body fluids, serum holds good for repeated evaluation of disease status and less invasive. Hence, analyzing the levels of serum aluminum, calcium, copper, and magnesium along with the gene expression may be useful in detecting Parkinson’s disease.

## Methods

### Data collection and protein interaction

The proteins containing metal as cofactors were retrieved from the protein databases (http://www.rcsb.org, and http://metalweb.cerm.unifi.it). Among the collected metal binding proteins, the membrane proteins were excluded due its unbounding of metal at their functional active site involving controlling membrane potential and ionic strength. Using the UniProt mapping tool (www.uniprot.org), each protein ID was converted into official protein symbol. Further, the collected metal-binding proteins were grouped based on their bounded metal. The protein with multi-metal was assigned to every group of its bounded metal. Twenty-four metal groups were retained containing a minimum of ten proteins in each group. The metal protein network (_metal_PN) was constructed using Cytoscape software for each metal group by extending each metal-binding protein to immediate neighbor interacting protein.

### Principle component analysis based on network properties

To determine the biological significance of 24 _metal_PNs, the 24 randomized, and 24 molecular pathway networks were generated. Each randomized network was constructed using Erdos–Renyi model. Twenty-four randomized networks were created containing an equal number of nodes corresponding to every 24 _metal_PNs. Similarly, the 24 molecular pathway networks were constructed by collecting proteins from the 24 randomly selected molecular pathways available in the KEGG database. Seven topological parameters were determined for each network using Cytoscape network analyzer. Principle component analysis (PCA) was performed using R-program, on the variables describing the topological parameters of 72 networks to group them based on their network properties^35^.

### Clustering, text-mining, and meta-analysis

ClusterONE algorithm was implemented on each _metal_PN to decompose large network into dense protein hubs^36^. Each protein hub represents the core functional component of _metal_PN. The top-ranked hub of each _metal_PN was selected to determine its involvement in PD. For which, the text-mining approach was carried out to collect the proteins reported in PD from literature and mapped to each top-ranked hub of 24 _metal_PN to select PD hubs.

### Text-mining

Indigenous R-code was developed to retrieve the abstract for the key terms related to Parkinson’s disease in conjunction with “Homo sapiens” from the PubMed database, published between the years 1990–2017. The collected 84,214 abstracts were examined for the presence of 22,853 protein symbols from the dictionary constructed by integrating various proteome databases^37–39^. The R-code looks for the co-occurrence of the terms “Parkinson’s disease” and protein symbol. We weigh the associations of protein with the PD based on the occurrence of each protein symbol and PD in the abstract based on the point-wise mutual information method. Further, the frequencies of the collected PD proteins across the abstracts were evaluated to have its presence in a minimum of three abstracts. The identified PD proteins were mapped to the top-ranked hub of each metal protein network (_metal_PN) to select PD associated hub.

### Meta-analysis

To determine the regulation of selected hub in PD, the expression of proteins encoding genes in the each hub was subjected to the meta-analysis. For which, the microarray gene expression datasets were extracted by two independent reviewers (AA & JS) from NCBI Gene Expression Omnibus GEO (http://www.ncbi.nlm.nih.gov/geo/) and Arrayexpress (https://www.ebi.ac.uk/arrayexpress/) using the keyword related to Parkinson’s disease. The inclusion criteria were set for the selection of dataset include (1) human case-control study, (2) dataset comparing PD with other neurological disease and healthy control, (3) study conducted in brain and/or blood tissue, (4) availability of raw or processed dataset. Also, we exclude the expression dataset of (1) non-human study, (2) in vivo study, (3) integrated and secondary analysis of expression data, and (4) the microarray dataset with less than two replicate samples per type. Any discrepancy in collected dataset was resolved by the third reviewer (SSJ), while the group discussion. The Preferred Reporting Items for Systematic Reviews and Meta-Analysis (PRISMA) guidelines published in 2015 was followed in the selection of datasets for this meta-analysis^40^.

### Meta-analysis of genes in _metal_PN hubs

The collected microarray datasets were subjected to INMEX^41^, and the analysis was carried out by following the procedure of Jose et al.^42^. In brief, each dataset was annotated by converting probe ID to Entrez ID. The gene intensity was log-transformed and limma processed to determine the significant genes *P*-value (≤ 0.05) dataset between control and PD. For meta-analysis, the datasets were merged to apply combat to decrease the batch effect. Further, Fisher’s statistical approach was used to combine significant *P*-values of each dataset to determine the significant genes from the merged datasets. For the collected 14 brain datasets, the meta-analysis was conducted to determine the significant genes in PD with *P*-value ≤ 0.05. Similarly, the significant PD genes in blood were identified from the merged four datasets.

### Classification algorithm to classify _metal_PNs hubs

Data classification was performed using Weka software (Waikato Environment for Knowledge Analysis)^43^. Thirty-four distinct classification algorithms were used to classify each hub into PD from normal based on the merged expression data of the meta-analysis. The protein of each hub was considered as features with its corresponding gene expression value as input for classification algorithm. The performance was evaluated based on the ten-times, ten-fold cross-validation technique. The threshold independent parameters such as sensitivity, specificity, accuracy and Matthews’s correlation coefficient were calculated for each hub. Further, top five hubs showing high classification accuracy between the 34 algorithms were selected and their proteins were subjected to KEGG pathway enrichment analysis^44^. For validation, the hub relating metal was analyzed using AAS in serum of control and PD. Simultaneously, the proteins in each hub was functionally prioritized using ToppGene tool^45^. In ToppGene tool, the text-mined 2088 PD proteins excluding hub proteins were inputted as training-set and the proteins of each hub was considered as test-set, to identify the functionally importance protein in each hub. Further, the change in expression of these proteins encoding genes were tested in peripheral blood mononuclear cell (PBMC) using Real time qPCR along with the previously known antioxidant (Superoxide dismutase; SOD1) and inflammatory markers (C-reactive protein; hsCRP) of PD.

### Participant selection

The participants were recruited at the National Neuroscience Center (NNC) and Nil Ratan Sircar Medical College and Hospital (NRS), Kolkata, India. The protocol of this study, including sample collection, processing and analysis was approved by the Research Ethics Boards Committee of the participating centers (NNC: Ref.No.Ath/2013) and NRS: Ref.No.25062012). The written informed consent was obtained from all participants prior to sample collection. The PD patients were enrolled and diagnosed by the neurologist based on neurological examination and medical history. To have a true positive PD patients, Unified Parkinson’s Disease Rating Scale (UPDRS) and Hoehn and Yahr scale were followed. For the comparative analysis, the participants free from neurological or neuropsychiatric disorders were taken and underwent neurological examinations similar as the patient for confirmation. Additionally, the selected controls are equally matched with the PD participants based on their age, gender, co-morbidities such as diabetes, hypertension, and cardio vascular disease (Supplementary Table 8).

### Inclusion and exclusion criteria

Participants for this study were selected by following the inclusion and exclusion criteria. Inclusion criteria: (1) ability to comply with study procedures. (2) participants aged 45–65 years. (3) body mass index (BMI) of 18–35 kg/m^2^. For participants with PD: (1) drug naïve, (2) presence of cardinal symptoms, (3) absence of secondary Parkinsonism because of drugs, (4) no features of prominent oculomotor palsy, cerebellar signs, vocal cord paresis, pyramidal signs, severe orthostatic hypotension, amyotrophy, or apraxia. The exclusion criteria include: (1) history of alcohol abuse or consumption, (2) smoker or tobacco user, (3) previous history of severe systemic diseases, (4) intake of minerals and chelating agents, and (5) acute infections, traumata or surgery in the last one year.

### Sample collection and processing procedure

The CSF and blood were drawn from the same patients and compared with healthy controls. The demographic and clinical characteristics of participants were recorded (see Supplementary Table 8). The CSF (control = 42; PD = 42) and peripheral blood (control = 87; PD = 87) samples were obtained from the participating centers. A few did not show willingness to provide CSF samples. Hence, CSF obtained only from 42 control and 42 PD by following the BioMS-eu consortium guidelines. Ten ml of CSF was obtained by lumbar spinal tap using a traumatic needle. The CSF sample was then centrifuged at 1200 rpm for 10 min at room temperature to collect cell-free supernatant. Similarly, blood (10 + 3 mL) was collected, 10 mL were centrifuged for 10 min at 3000 rpm to separate serum and the remaining 3 mL was used for PBMC isolation. The separated samples were stored at −80 °C for further analysis.

### Analysis of hub associated metals in serum and CSF

All precautions were taken following the NCCLS Guidelines to avoid contaminants while sample collection and processing for the element analysis. Nitric acid based microwave digestion was carried out^46^. For calibration, NIST SRM 3100 series single-element standard solution was used at various concentrations to have standard graphs. Also, the limits of detection (LoDs) for the metals were determined by analyzing blank solutions. The element concentration of aluminum and iron in serum was analyzed using electrothermal (AAS SHIMADZU AA-6200, Kyoto, Japan). Whereas, calcium, copper, and magnesium were analyzed using flame AAS VARIAN AA-240, Varian Inc, (USA). The internal standards were run between every six samples to determine the accuracy and quality of the analysis.

### Gene expression analysis of clusters proteins, antioxidant, and inflammatory marker

Peripheral blood mononuclear cell were isolated using Histopaque-1077 (Sigma-Aldrich) according to the manufacturer’s protocol. Further, the TRIzol (Invitrogen) reagent was used for isolation of total RNA from PBMC. The isolated RNA was quantified using Nanodrop 2000 (Thermo Scientific) to construct cDNA from total RNA^47^. Quantitative real-time PCR was performed to determine the expression of protein encoding gene in each metal hub and antioxidant and inflammatory marker between PD and control. The expression analysis was executed using ABI-7000 (Applied Biosystems) with SYBR green master mix (Takara) and gene specific primers (see Supplementary Table 7). The expression of targeted gene was normalized with β-actin housekeeping gene and relative expression was calculated following the 2^−ΔΔCt^ method.

### Statistical analysis

The statistical analysis was performed using SPSS software. The student *t*-test was performed to determine the statistical difference (*P* ≤ 0.05) between the control and PD for the measured metals and PBMC gene expression, respectively. Further, correlation analysis was carried out to determine the interdependency of (1) Metal concentration between serum and CSF, (2) PBMC gene expression with serum metal concentration, (3) PBMC SOD expression with metal and metal hub protein, and (4) PBMC hsCRP expression with metal and metal hub protein.

### Reporting summary

Further information on research design is available in the Nature Research Reporting Summary linked to this article.

## Supplementary information

Supplementary Information

Reporting Summary

## Data Availability

The raw gene expression data used in this study were obtained from the NCBI-GEO DataSets (https://www.ncbi.nlm.nih.gov/gds). The gene expression dataset can be accessed using accession IDs GSE7621, GSE8397, GSE19587, GSE20141, GSE20146, GSE20163, GSE20164, GSE20168, GSE20186, GSE20291, GSE20292, GSE20295, GSE28894, and GSE49036 for brain. Similarly for blood, GSE6613, GSE22491, GSE54536, and GSE72267 can be used.

## Code availability

IBM SPSS package version 24.0 was used for the statistical analysis. The meta-analysis based gene expression was executed using integrative meta-analysis of gene expression (INMEX). The indigenous R-code was used for text-mining will be provided on request.
