# Supplementary material for: Interdependence of metals and its binding proteins in Parkinson’s disease for diagnosis
Source: NPJ Parkinsons Dis. 2021 Jan 4;7:3. doi: 10.1038/s41531-020-00146-7 (PMC7782529; doi:10.1038/s41531-020-00146-7)
Supplement: Supplementary file 1 — Supplementary Information [file 41531_2020_146_MOESM1_ESM.pdf]

Supplementary Table 1. Seven important topological parameters were determined for each network. The values of each parameter were represented as mean ± standard deviation of 24 networks across $_{metal}$PN, randomized and molecular pathway network.

**Topological Parameter**	**Networks (mean ± SD)**		
	**$_{metal}$PNs**	**Randomized networks**	**Molecular pathway networks**
Average Shortest Path Length	4.35±0.86	7.11±1.71	2.339± 0.38
Betweenness Centrality	0.0059±0.01	0.019±0.02	0.019±0.01
Closeness Centrality	0.26±0.06	0.151±0.03	0.318±0.06
Clustering Coefficient	0.063±0.04	0.0033±0.01	0.298±0.09
Degree	4.695±0.78	2.648±0.99	7.157±2.54
Neighborhood Connectivity	1330.57±259.51	3.301±1.14	10.67±4.29
Topological Coefficient	0.099±0.05	0.235±0.014	0.2617±0.048

Supplementary Table 2. The table contain list of pathways regulated by protein in all five $_{metal}$PN clusters.

Metal	Pathways
Aluminium	Maturity onset diabetes of the young
Calcium	AGE RAGE signaling pathway in diabetic complications
Calcium	Apoptosis pathway
Calcium	B cell receptor signaling pathway
Calcium	Biosynthesis of amino acids
Calcium	C type lectin receptor signaling pathway
Calcium	cAMP signaling pathway
Calcium	Carbon metabolism
Calcium	Cholinergic synapse
Calcium	Circadian entrainment
Calcium	Complement and coagulation cascades
Calcium	Dopaminergic synapse
Calcium	Endocrine resistance
Calcium	Endocytosis
Calcium	Epithelial cell signaling in Helicobacter pylori infection
Calcium	ErbB signaling pathway
Calcium	Estrogen signaling pathway
Calcium	GABAergic synapse
Calcium	Glycine, serine and threonine metabolism
Calcium	Glycosphingolipid biosynthesis
Calcium	Glyoxylate and dicarboxylate metabolism
Calcium	GnRH signaling pathway
Calcium	IL 17 signaling pathway
Calcium	Leukocyte transendothelial migration
Calcium	MAPK signaling pathway
Calcium	Metabolic pathways
Calcium	mRNA surveillance pathway
Calcium	mTOR signaling pathway
Calcium	Neuroactive ligand receptor interaction
Calcium	Neurotrophin signaling pathway
Calcium	NOD like receptor signaling pathway
Calcium	Oxytocin signaling pathway
Calcium	Pathways in cancer
Calcium	Pertussis
Calcium	Prolactin signaling pathway
Calcium	Ras signaling pathway
Calcium	Relaxin signaling pathway
Calcium	Retrograde endocannabinoid signaling
Calcium	Rheumatoid arthritis
Calcium	Ribosome biogenesis in eukaryotes
Calcium	RNA transport
Calcium	Synaptic vesicle cycle

Calcium	T cell receptor signaling pathway
Calcium	Th1 and Th2 cell differentiation
Calcium	Th17 cell differentiation
Calcium	Tight junction
Calcium	TNF signaling pathway
Calcium	Toll like receptor signaling pathway
Calcium	Transcriptional misregulation in cancer
Calcium	Wnt signaling pathway
Copper	Amoebiasis
Copper	Antigen processing and presentation
Copper	Hematopoietic cell lineage
Copper	Tight junction
Iron	Adrenergic signaling in cardiomyocytes
Iron	AGE RAGE signaling pathway in diabetic complications
Iron	Apelin signaling pathway
Iron	Apoptosis
Iron	B cell receptor signaling pathway
Iron	cAMP signaling pathway
Iron	Cellular senescence
Iron	cGMP PKG signaling pathway
Iron	Cholinergic synapse
Iron	Circadian entrainment
Iron	Cortisol synthesis and secretion
Iron	Cushing syndrome
Iron	Cysteine and methionine metabolism
Iron	Dopaminergic synapse
Iron	Endocrine resistance
Iron	ErbB signaling pathway
Iron	Estrogen signaling pathway
Iron	Glucagon signaling pathway
Iron	GnRH signaling pathway
Iron	IL 17 signaling pathway
Iron	Long term potentiation
Iron	Longevity regulating pathway
Iron	MAPK signaling pathway
Iron	Metabolic pathways
Iron	Natural killer cell mediated cytotoxicity
Iron	Neurotrophin signaling pathway
Iron	NOD like receptor signaling pathway
Iron	Non alcoholic fatty liver disease (NAFLD)
Iron	Osteoclast differentiation
Iron	Oxidative phosphorylation
Iron	Oxytocin signaling pathway
Iron	Pertussis
Iron	PI3K Akt signaling pathway

Iron	Prolactin signaling pathway
Iron	Protein processing in endoplasmic reticulum
Iron	Regulation of actin cytoskeleton
Iron	Relaxin signaling pathway
Iron	Ribosome
Iron	RNA transport
Iron	Sphingolipid metabolism
Iron	Sphingolipid signaling pathway
Iron	Spliceosome
Iron	T cell receptor signaling pathway
Iron	Th1 and Th2 cell differentiation
Iron	Thermogenesis
Iron	Thyroid hormone synthesis
Iron	Tight junction
Iron	TNF signaling pathway
Iron	Toll like receptor signaling pathway
Iron	Wnt signaling pathway
Magnesium	Apelin signaling pathway
Magnesium	Apoptosis
Magnesium	EGFR tyrosine kinase inhibitor resistance
Magnesium	Insulin signaling pathway
Magnesium	MAPK signaling pathway
Magnesium	mTOR signaling pathway
Magnesium	PI3K
Magnesium	T cell receptor signaling pathway
Magnesium	TNF signaling pathway

Supplementary Table 3. Correlation of each metal representing the inter dependency of serum-CSF in control and PD. Significant change in association of metal concentration between serum and CSF was noticed for aluminum, calcium, copper and magnesium in PD.

Metal	R value	Statistical significance between the slopes (p-value)
Aluminum	0.384	0.01*
Calcium	0.3172	0.04*
Copper	0.342	0.026*
Iron	-0.1012	0.52
Magnesium	-0.3242	0.036*

*Statistical significance (P-value ≤ 0.05)

Supplementary Table 4. The correlation between serum metal concentration and gene expression of each hub showing significant change in interdependency for analyzed metal-gene expression pair in PD compared to control.

Metal	Gene	R value	Statistical significance between the slopes (p-value)
Aluminum	EFEMP2	0.9997	0.015594
Calcium	MMP9	0.9972	0.0476
Copper	B2M	-0.9993	0.028473
Iron	MEAF2A	-0.999215	0.0284
Magnesium	TARDBP	0.978439	0.0216

Statistical significance (P-value $\leq$ 0.05)

Supplementary Table 5. The correlation between serum metal concentration with antioxidant and inflammatory marker which shows significant change in interdependency for analyzed metal-SOD1 & hsCRP pair in PD compared to control

Metal	Antioxidant & Inflammatory marker	R value	Statistical significance between the slopes (p-value)
Aluminum		-0.99890	0.04027
Calcium		-0.99833	0.0402
Copper	SOD1	-0.99784	0.049325
Iron		-0.999988	0.02847
Magnesium		-0.998	0.04027
Aluminum		0.99971	0 .015594
Calcium		0.997771	0.04318
Copper	hsCRP	-0.999977	0.028473
Iron		-0.99873	0.04027
Magnesium		0.9982273	0.038203

Statistical significance (P-value ≤ 0.05)

Supplementary Table 6. Correlation between the metal hub protein with SOD1 and hsCRP showed significant change in interdependency when compared with PD to control.

Metal hub protein	Antioxidant & Inflammatory marker	R value	Statistical significance between the slopes (p-value)
EFEMP2		-0.999	0.028
MMP9		-0.99993	0.02847
B2M	SOD1	0.99717	0.048495
MEAF2A		0.99835	0 .037126
TARDBP		-0.9999	0.02847
EFEMP2		0.999711	0.015594
MMP9		0.999647	0.01800
B2M	hsCRP	0.99902	0.028473
MEAF2A		0.999154	0.027012
TARDBP		0.999312	0.023822

Statistical significance (P-value ≤ 0.05)

Supplementary Table 7. Gene expression primer for the protein encoded gene of each metal hub used in qRT-PCR

GENE	SEQUENCES	
B2M	Forward	TACTACACTGAATTCACCCCC
	Reverse	TTCAAACCTCCATGATGCTG
TARDBP	Forward	CTTATGGTGCAGGTCAAGAAAG
	Reverse	CTTTTCTGCTTCTCAAAGGCTC
MEF2A	Forward	CTCCACTAAATACCCAGAGGAT
	Reverse	AGCGCTGGTCAGTGAATAAT
EFEMP2	Forward	GCTACACGGAATGCACAGAT
	Reverse	AGTGGTTGATGCACTTCATTTC
MMP9	Forward	GACAAGCTCTTCGGCTTCTG
	Reverse	ACAAACTGTATCCTTGGTCCG
GAPDH	Forward	AAGGTGAAGGTCGGAGTCAA
	Reverse	ACATGTAAACCATGTAGTTGAGGT
SOD1	Forward	ACTGGTGGTCCATGAAAAAGC
	Reverse	AACGACTTCCAGCGTTTCCT
hsCRP	Forward	ATTCAGGCCCTTGTATCACTG
	Reverse	TCCGACTCTTTGGGAAACAC

Supplementary Table 8. Characteristics of the research participants involved in this study

Parameter	PD (n=87)	Controls(n=87)
Age(Year)	53±8	55±6
Gender (Male/ Female)	43/44	43/44
Body mass index	28±3	30± 3
Onset(Year)	0.6±0.1	Healthy
H & Y stages	All PD belongs to stage 1	Healthy
UPDRS score	29.92±6.7	Healthy
**Percentage of participant presenting comorbidity**		
Diabetes (%)	20.68	18.40
Cardio Vascular Disease (%)	9.19	10.34
Hypertension (%)	12.64	14.95
**Percentage of participant presenting cardinal symptoms**		
Rigidity (%)	75.86	No symptoms observed
Resting tremor (%)	78.16	No symptoms observed
Bradykinesia (%)	57.47	No symptoms observed

Unified Parkinson's Disease Rating Scale(UPDRS) ; Hoehn & Yahr (H& Y) stage

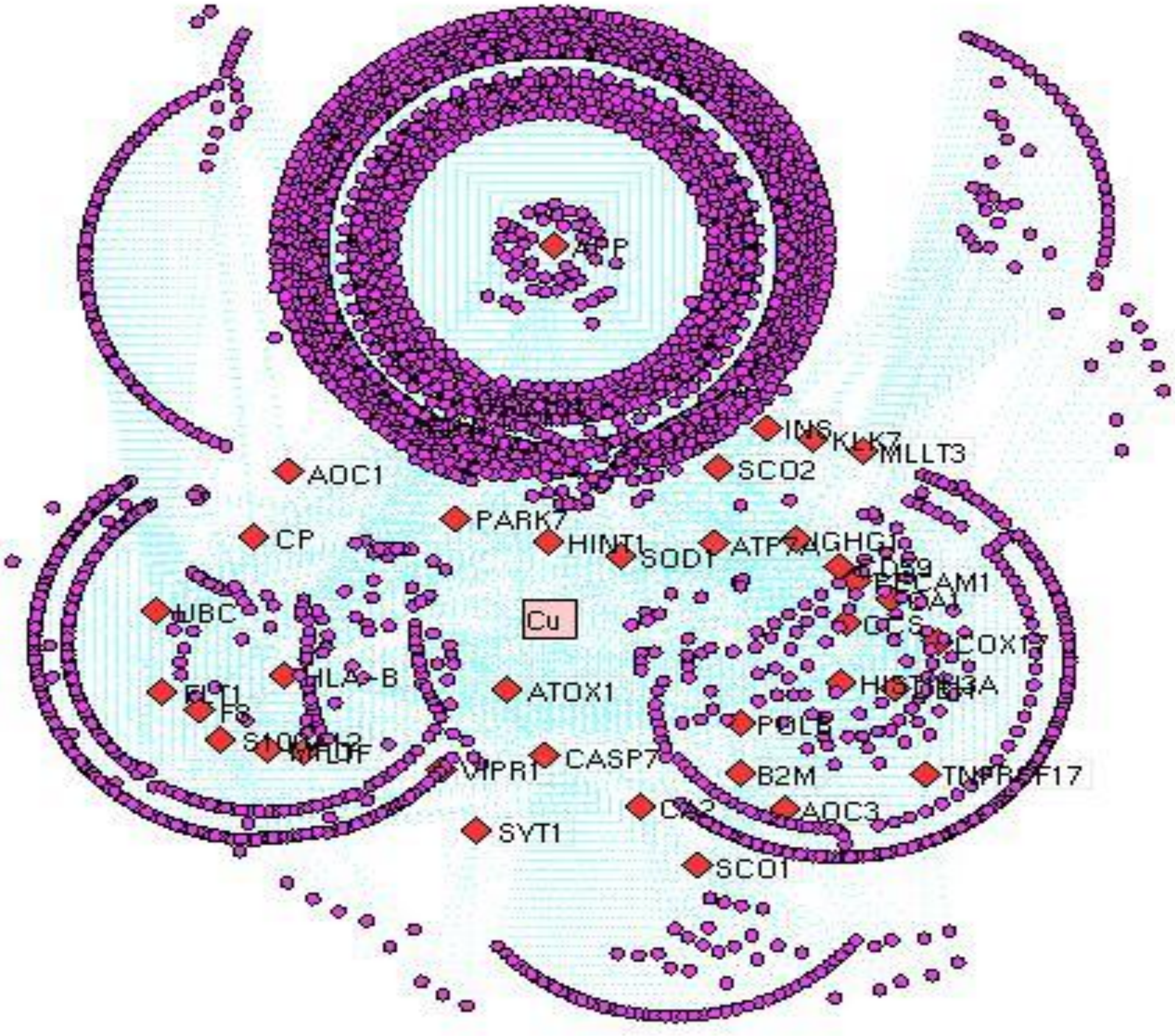

Supplementary Figure 1: Network representing copper $_{metal}$PN with 4384 interacting proteins, red color node indicates copper-binding proteins; magenta indicates its interacting protein.

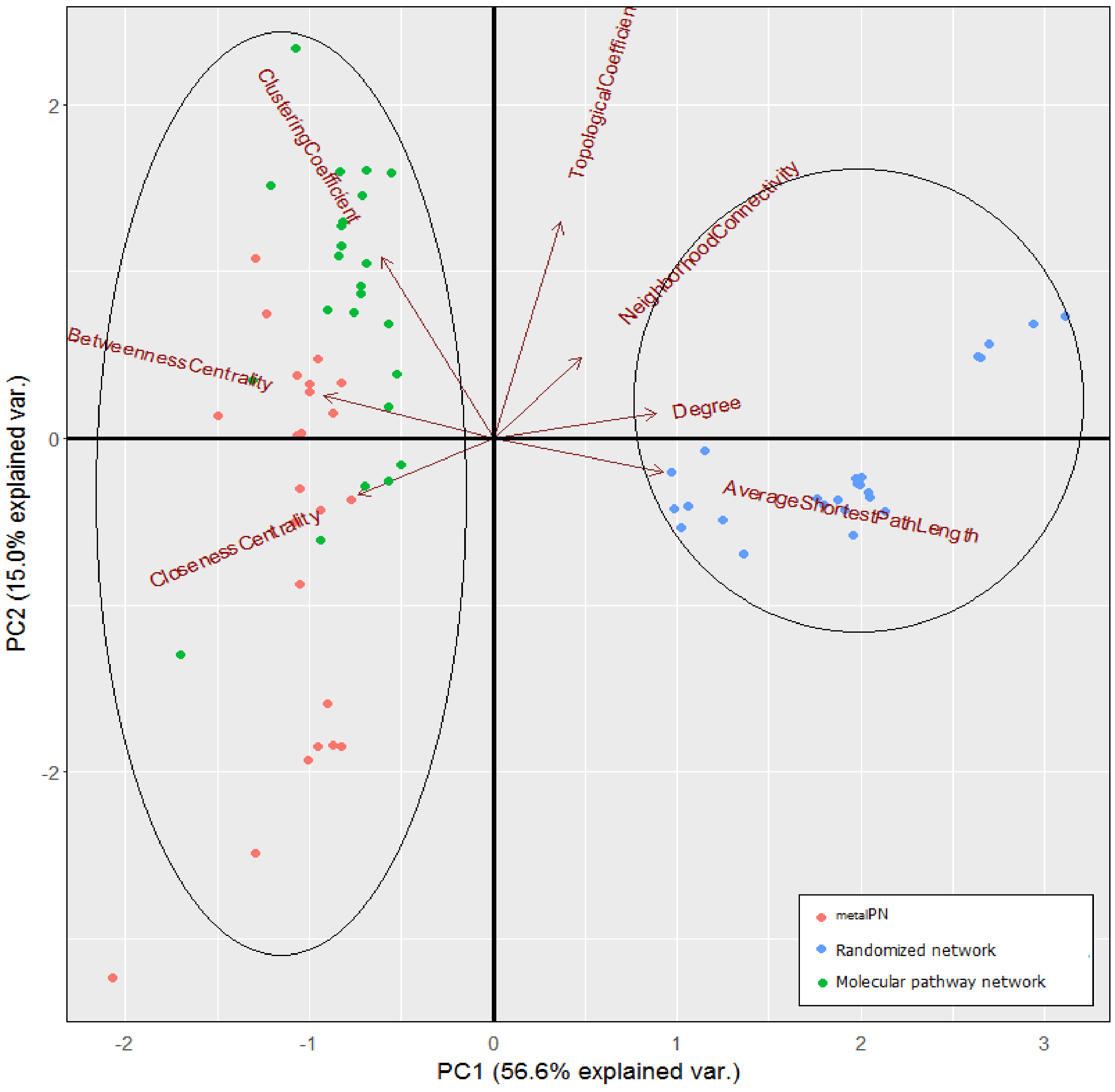

Supplementary Figure 2: The PCA plot based on topological properties describing the grouping of $_{metal}$PNs (red), molecular pathway network (green) and randomized network (blue). The first two components (PC1 and PC2) explained 71.6% of variance.

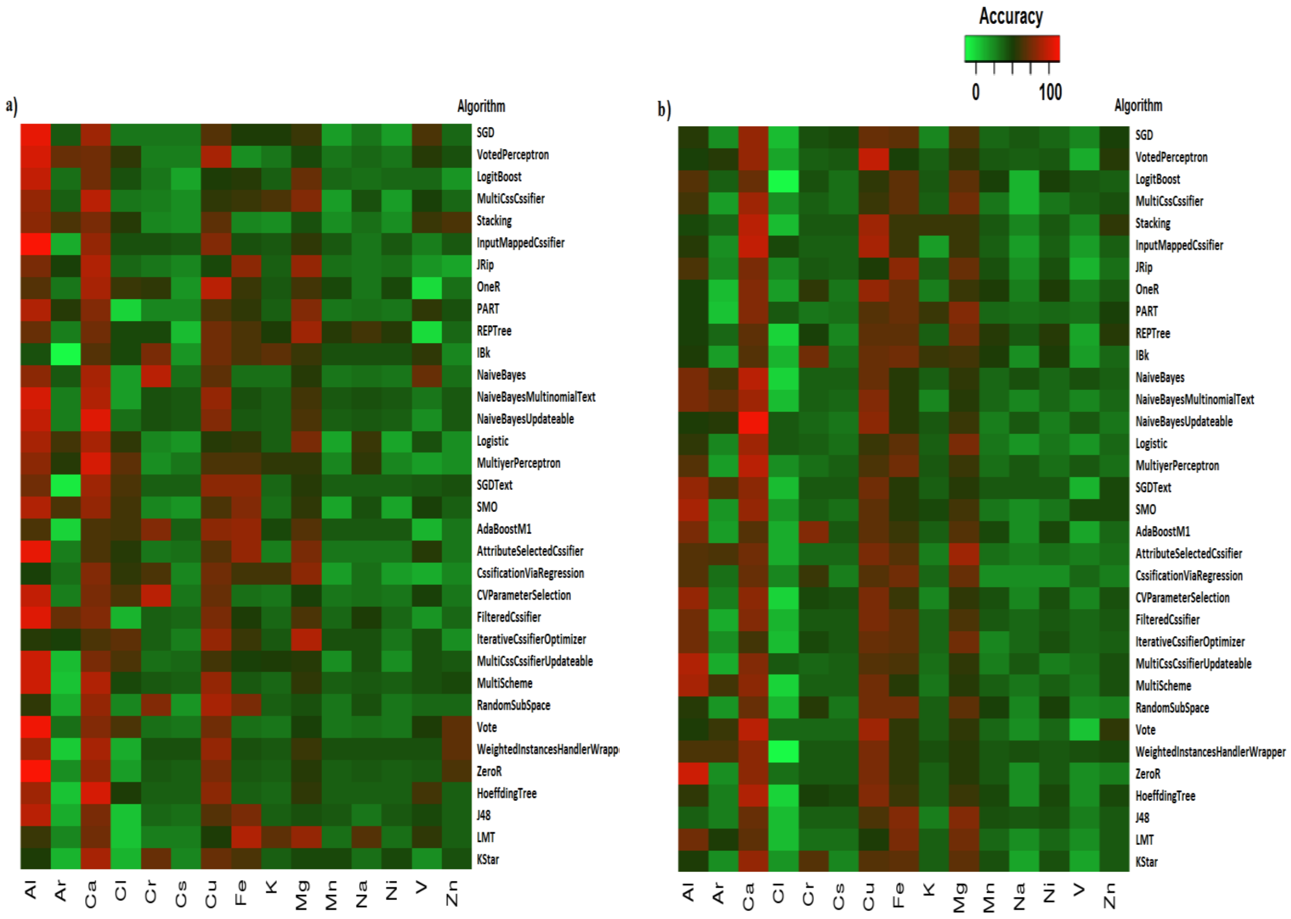

Supplementary Figure 3: Heat map shows the predicted accuracy of each metal hub in Blood (a) and Brain (b) using 34 distinct neural network algorithms. The color gradient indicates low accuracy (green) and high accuracy (red) describing the predicted accuracy of PD from normal.

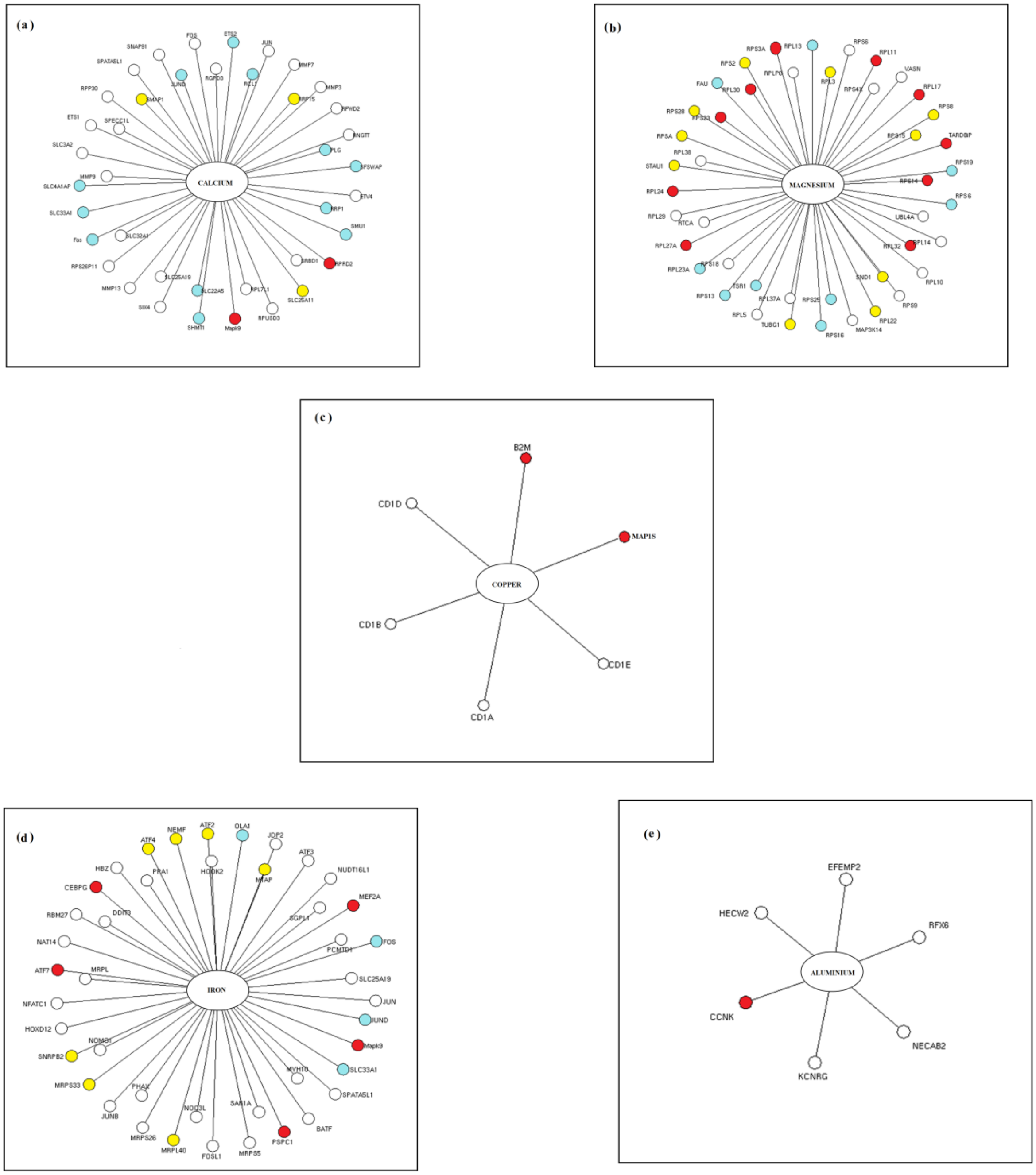

Supplementary Figure 4: Five significant hubs, a) Calcium, b) Magnesium, c) Copper, d) Iron, and e) Aluminium showing the differential expressed Parkinson's disease genes in brain tissue colored as blue, blood (yellow) and common in both tissue (red).

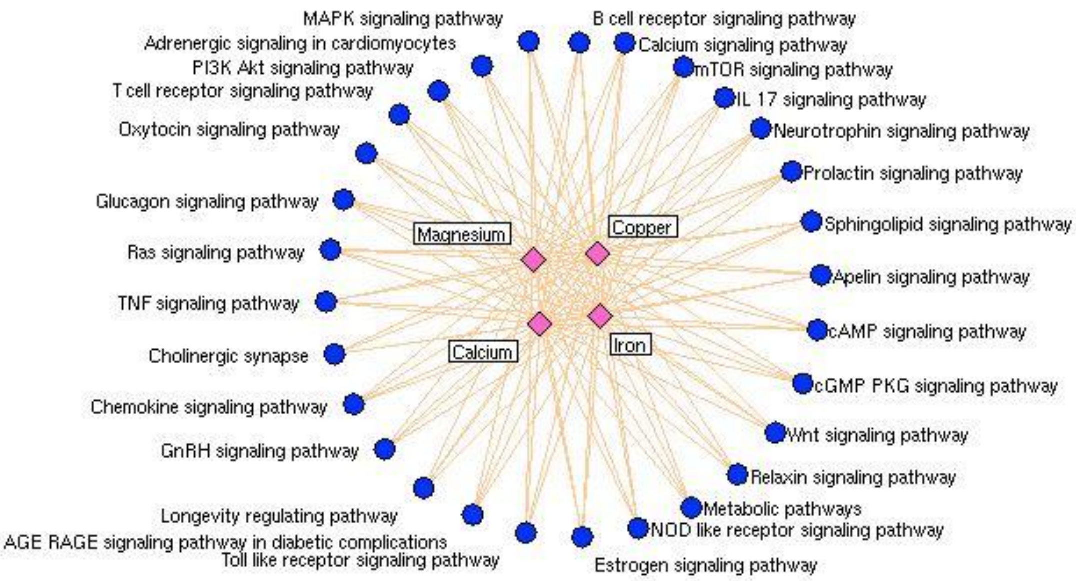

Supplementary Figure 5: Commonly over-represented 29 pathways related to proteins of calcium, magnesium, copper, and iron hubs.
